# Healthy rabbits are susceptible to Epstein-Barr virus infection and infected cells proliferate in immunosuppressed animals

**DOI:** 10.1186/s12985-015-0260-1

**Published:** 2015-02-18

**Authors:** Gulfaraz Khan, Waqar Ahmed, Pretty S Philip, Mahmoud H Ali, Abdu Adem

**Affiliations:** Departments of Microbiology and Immunology, Faculty of Medicine and Health Sciences, United Arab Emirates University, Al Ain, PO 17666, United Arab Emirates; Pharmacology, Faculty of Medicine and Health Sciences, United Arab Emirates University, Al Alin, United Arab Emirates

**Keywords:** EBV, Rabbit model, Immunosuppression, EBV-mediated proliferation

## Abstract

**Background:**

Epstein-Barr virus (EBV) is an oncogenic virus implicated in the pathogenesis of several human malignancies. However, due to the lack of a suitable animal model, a number of fundamental questions pertaining to the biology of EBV remain poorly understood. Here, we explore the potential of rabbits as a model for EBV infection and investigate the impact of immunosuppression on viral proliferation and gene expression.

**Methods:**

Six healthy New Zealand white rabbits were inoculated intravenously with EBV and blood samples collected prior to infection and for 7 weeks post-infection. Three weeks after the last blood collection, animals were immunosuppressed with daily intramuscular injections of cyclosporin A at doses of 20 mg/kg for 15 days and blood collected twice a week from each rabbit. The animals were subsequently sacrificed and tissues from all major organs were collected for subsequent analysis.

**Results:**

Following intravenous inoculation, all 6 rabbits seroconverted with raised IgG and IgM titres to EBV, but viral DNA in peripheral blood mononuclear cells (PBMCs) could only be detected intermittently. Following immunosuppression however, EBV DNA could be readily detected in PBMCs from all 4 rabbits that survived the treatment. Quantitative PCR indicated an increase in EBV viral load in PBMCs as the duration of immunosuppression increased. At autopsy, splenomegaly was seen in 3/4 rabbits, but spleens from all 4 rabbit were EBV PCR positive. EBER-*in situ* hybridization and immunoshistochemistry revealed the presence of a large number of EBER-positive and LMP-1 positive lymphoblasts in the spleens of 3/4 rabbits. To a lesser extent, EBER-positive cells were also seen in the portal tract regions of the liver of these rabbits. Western blotting indicated that EBNA-1 and EBNA-2 were also expressed in the liver and spleen of infected animals.

**Conclusion:**

EBV can infect healthy rabbits and the infected cells proliferate when the animals are immunocompromised. The infected cells expressed several EBV-latent gene products which are probably driving the proliferation, reminiscent of what is seen in immunocompromised individuals. Further work is required to explore the potential of rabbits as an animal model for studying EBV biology and tumorigenesis.

## Introduction

Epstein-Barr virus (EBV) is a human lymphotropic herpesvirus implicated in the pathogenesis of a number of malignancies of both epithelial and lymphoid origin, including Burkitt’s lymphoma (BL), nasopharyngeal carcinoma (NPC), post-transplant lymphoproliferative disease (PTLD) and Hodgkin lymphoma (HL) [1]. A large part of our current understanding of the biology of EBV comes from studies of *in vitro* infection of human B-lymphocytes. Infection of B-cells leads to their immortalization [2]. In these cells, the virus establishes type III latency in which up to 11 viral products, namely 6 Epstein-Barr nuclear antigens (EBNA-1, EBNA-2, EBNA-3a, EBNA-3b, EBNA-3c, EBNA-LP), three virus-encoded latent membrane proteins (LMP-1, LMP-2a, LMP-2b) and two non-protein encoding RNAs (EBER-1 and EBER-2) are expressed without killing the cell [3,4]. Although the mechanism(s) by which EBV causes cell immortalization is not clear, it has been shown that some of these EBV latent proteins influence, directly or indirectly, a number of key cellular processes, including inhibition of apoptosis, induction of cell proliferation and transformation [5-8].

In contrast to *in vitro* infection, the biology of EBV infection *in vivo* is much more complex and less well understood. The virus is widespread in all human populations, with over 90% of adults worldwide being infected [1]. Although it is well known that EBV is transmitted via the oral route, it is unclear whether B-cells or oropharyngeal squamous epithelial cells are the initial sites of infection. Ironically, even in acute infections where there is abundant viral presence, only B-cells and not epithelial cells have been shown to be infected [9-11]. More recent studies suggest that EBV-infected B-lymphocytes can transfer EBV to epithelial cells by close interaction between the two cell types [12,13]. However, the identity of the virus-producing cells responsible for the infectious virus present in the saliva [14] remains in doubt. What is clear is that EBV establishes a life-long persistence in resting memory B-lymphocytes [15,16]. The frequency of these cells is tightly regulated in the healthy individuals [17] and probably evade the host immune response by down-regulating essential cellular activation molecules and limiting viral gene expression to one or two proteins only [18,19]. Disruption of this tightly regulated system, as seen in allograft recipients receiving immunosuppressive therapy, can lead to EBV-driven lymphoproliferative disorders (PTLD) [20-23]. In these patients, the frequency of circulating EBV-infected cells increases dramatically soon after transplantation and this increase correlates with the development of B-cell lymphoproliferations [24-26]. However, the precise molecular pathways taken by EBV-infected cells on their route to the development of EBV-associated PTLD remains to be demonstrated. One major obstacle which has hampered research in unraveling the biology of EBV and its role in the pathogenesis of EBV-associated diseases has been the lack of a suitable animal model. Humans are the only natural host for EBV. EBV is highly cell tropic, infecting only human B-cells expressing CD21 receptor [27]. B-cells from animals such as mice or rats cannot be infected with EBV, *in vivo* or *in vitro*. EBV-like herpesviruses infecting mice [28,29] or primates [30,31] have been used to study EBV, as have humanized mice [32,33]. These models, albeit useful, have their drawbacks and do not fully represent natural bona fide EBV infection in humans. Thus, without a suitable animal model, a number of fundamental questions about the biology of EBV and its role in the pathogenesis of human diseases remain outstanding.

Recently, a Japanese group has successfully infected rabbits with EBV and shown that the virus can persist in these animals for several years without any major pathologies [34-36]. Moreover, it has been shown that EBV inoculation via intranasal and oral routes can also lead to persistent EBV infection [34,37]. If these findings can be independently confirmed, it could open up many channels for studying the biology of EBV and its link to the pathogenesis of EBV-associated human diseases. In this study, we show that rabbits can indeed be infected with EBV and further show that the infected cells proliferate under immunosuppressive conditions, similar to what has been described for allograft recipients on immunosuppressive drugs [21,38].

## Results

### Rabbits respond to EBV upon IV inoculation of the virus

**Figure 1:**
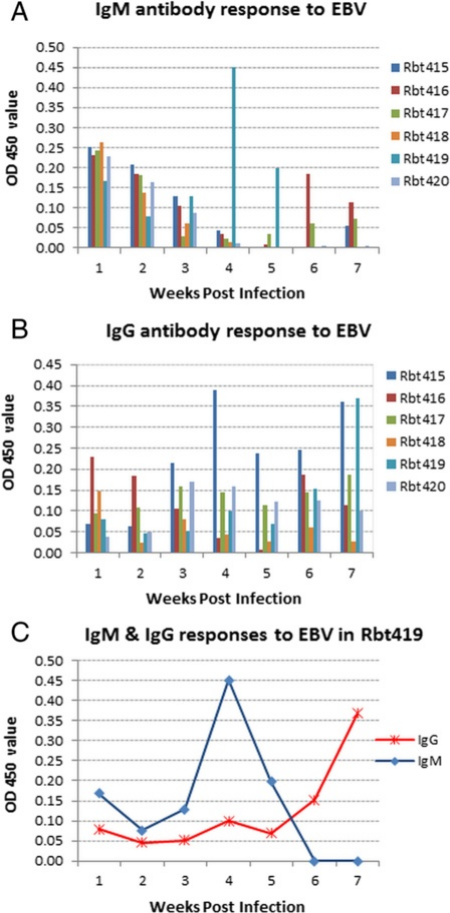
**Immune responses to EBV.** IgM **(A)** and IgG **(B)** responses to EBV infection in 6 healthy rabbits (Rbt 415–420) over a period of 7 weeks post infection. Generally, IgM levels were elevated in the first week post-infection and then tailed off by week 5. In contrast, IgG levels gradually increased and remained high up to the 7 weeks follow up. In one rabbit (Rbt 419), both the IgM and IgG responses to EBV were delayed compared to the other animals, peaking at week 4 for IgM and week 7 for IgG **(C)**. The data presented here is for plasma at dilution 1/100. Similar patterns were seen with plasma diluted 1/200 and 1/400, but with lower OD values.

Six healthy male New Zealand white rabbits aged 2–4 months (0.8-1.2 kg) were exposed to EBV by intravenous injection of 1 ml of cell-free, 0.45 μm filtered culture supernatant from B95-8 cell line. Each animal was inoculated 3 times on alternative days. The average EBV copy number in each inoculum, as determined by quantitative PCR (qPCR) [39] was estimated to be 1.74 × 10^7^ copies. The infectivity of the virus in the culture supernatant was assessed by its ability to successfully immortalize human PBMCs *in vitro* [40]. All 6 animals seroconverted and mounted a strong antibody response, but none developed any systemic signs of acute EBV infection. As expected, IgM was the first antibody to be triggered. In general, IgM levels were highest in week 1 and then gradually declined to background levels by week 5 (Figure 1A). IgG levels on the other hand were low to start with, but then increased and remained fairly high during the 7 weeks follow up (Figure 1B). All the OD readings were calculated relative to pre-infection plasma. Interestingly, in one rabbit (Rbt 419), both the IgG and IgM responses, albeit strong, were delayed compared to the other animals and peaked at weeks 4 for IgM and 7 for IgG (Figure 1C). The significance of this observation is not clear, however it is noteworthy that this animal subsequently developed an extensive and widespread EBV infection.

### Detection of EBV in peripheral blood of rabbits pre- and post-immunosuppression

**Figure 2:**
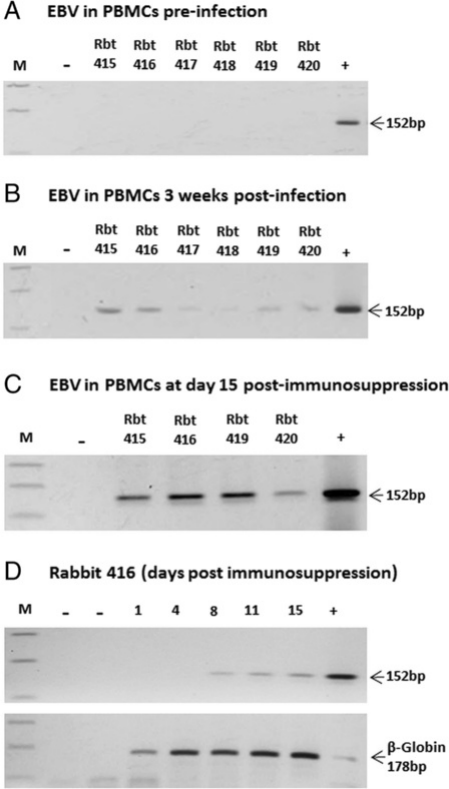
**Detection of EBV in rabbit PBMCs pre- and post-infection.** EBV BamH1 W PCR (amplicon size: 152 bp) was performed on DNA extracted from PBMCs collected from rabbits at various stages of infection: Pre-infection **(A)**; at week 3 post-infection **(B)** and at week 13 post-infection **(C)** (the last 15 days of which the animals were treated with daily injections of 20 mg/Kg of cyclosporin A (CsA) for 15 days). None of the animals were EBV positive prior to inoculation **(A)**. At week 3 post-infections, weak amplifications were seen in all 6 animals **(B)**. However, EBV positivity was intermittent and many of the samples taken at other time periods were negative. Following CsA treatment, all 4 rabbits that survived the 15-day CsA treatment were clearly EBV positive **(C)**. Furthermore, EBV positivity correlated with immunosuppression **(D)**. A representative data for rabbit 416 is provided as an example. EBV positivity was only seen about one week after the start of the immunosuppressive treatment. Positive (+) and negative controls (−) are indicated (see methods for further details).

For the detection of EBV, PCR was performed on DNA extracted from PBMCs of blood samples collected at weekly intervals post-infection from all 6 rabbits. As expected, no amplification signals were detected in any of the rabbits pre-infection (Figure 2A). However, 2–7 weeks after infection, weak amplification signals were detected intermittently in some samples in all 6 rabbits (Figure 2B). At week 10 post-infection, animals were treated with a daily dose of 20 mg/kg of cyclosporin A (CsA) for a period of 15 days [41,42]. One of the animals (Rbt 417) died overnight after the first CsA injection due to widespread fungal infection. A second animal (Rbt 418) was found to be very lethargic, not eating and generally looking unwell. This animal also had evidence of fungal infection. On the advice of the veterinarian and following the animal ethics guidelines, the animal was sacrificed before the second dose of CsA. Tissues from all major organs were collected from both rabbits. The remaining 4 animals survived the 15 day immunosuppressive treatment and we were able to collect blood samples at regular intervals. In contrast to pre-immunosuppression, PBMCs from blood samples taken post-immunosuppression gave strong amplification signals for EBV. This was particularly notable for blood samples taken on day 15 of treatment (Figure 2C). Viral load appeared to increase with increasing duration of immunosuppression (Figure 2D). This was then examined further using quantitative PCR (qPCR) (see ahead).

### Determination of EBV viral load and viral gene expression in peripheral blood of rabbits during immunosuppression

**Figure 3:**
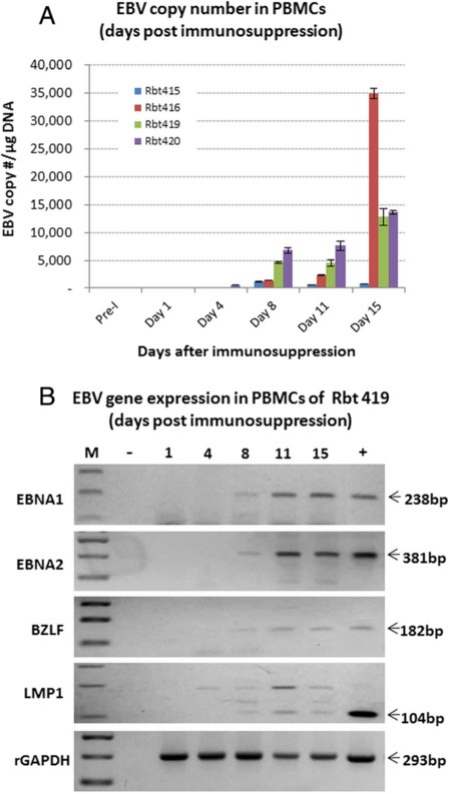
**Determination of viral load and viral gene expression following immunosuppression. A** EBV viral load in PBMCs (copies/μg DNA) was determined using qPCR. Three independent experiments were carried out for each sample at each interval post-cyclosporin A (CsA) treatment. The mean values, together with the standard deviations are presented. Viral load clearly increased with increasing duration of immunosuppression. No EBV was detected pre-infection (Pre-I) or in the first few days post-CsA treatment of EBV infected animals. **B** Reverse transcriptase PCR for EBV EBNA-1, EBNA-2, BZLF, and LMP-1 was performed on RNA extracted from PBMCs of rabbits at various time intervals following daily treatment with CsA. Weak and intermittent expression of several EBV genes was detected towards the end of the CsA treatment period. Positive (+) and negative (−) controls and the size of each amplicon are indicated.

Using qPCR and Namalwa cell line DNA as standards [39], we determined EBV viral load in PBMCs collected from rabbits during the 15 day CsA immunosuppressive treatment (Figure 3A). Analogous to post-transplant patients on immunosuppressive therapy, EBV viral load increased with increasing duration of immunosuppressive treatment [24,26]. The highest viral loads were noted in samples collected at day 15 of treatment. Prior to treatment, EBV was undetectable in 50 ng of PBMCs DNA that was used in each qPCR reaction (Figure 3A).

Analysis of EBV gene expression using reverse transcriptase PCR (RT-PCR) indicated that several EBV latent genes were expressed in PBMCs, notably towards the end of the 15 day CsA treatment period (Figure 3B). EBV gene expression was variable; EBNA-1 and EBNA-2 were clearly expressed by day 15 whilst the expression of BZLF and LMP-1 was at best weak and intermittent. The expression of the lytic marker BZLF, albeit intermittent, suggests that some lytic replication may be occurring during immunosuppression.

### Autopsy and macroscopic findings

**Figure 4:**
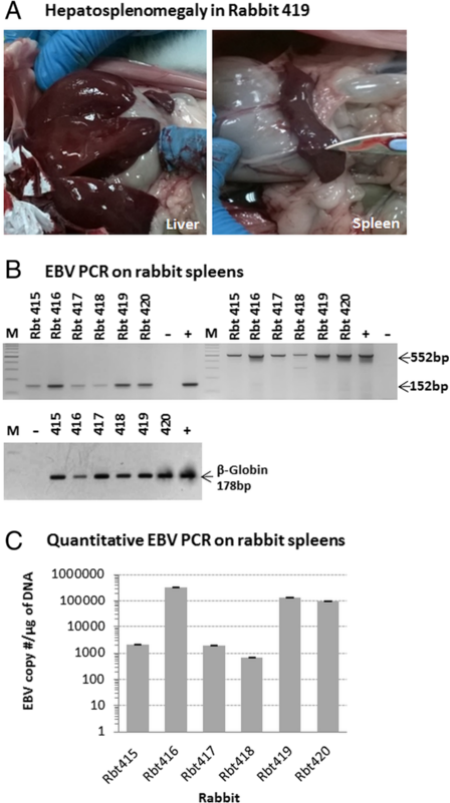
**Hepatosplenomegaly in rabbit 419.** Splenomegaly was noted at autopsy in 3 of the 4 rabbits that survived the 15 day cyclosporin A (CsA) treatment. In rabbit 419, both the spleen and liver were enlarged **(A)**. EBV BamH1 W PCR on DNA extracted from rabbit spleens indicated that all were EBV positive **(B)**. Amplification signals were stronger in the spleens of the 4 animals that survived the 15 day immunosuppressive treatment compared to the 2 animals (Rbt 417 and Rbt 418) that died on day 2 after the start of the CsA treatment. Similar results were observed using two different sets of EBV BamH1 W primers (amplicon size of 552 bp and 152 bp). Quantitative PCR analysis **(C)** confirmed the standard end-point PCR findings observed in Figure 4B.

The 4 rabbits that survived the 15-day CsA treatment were euthanized and sacrificed on day 17 and tissues from all major organs collected. At autopsy, splenomegaly was noted in 3 of the 4 rabbits. This was most prominent in rabbit 419, which also had hepatomegaly (Figure 4A). DNA extracted from spleen samples gave strong positive signals for EBV (Figure 4B). The signals were stronger for the 4 rabbits that survived the 15-day CsA treatment compared to the two rabbits (Rbt 417 and 418) that died early on during the treatment period. PCR for EBV was performed using 2 different sets of BamH1 W primers giving amplification products of 152 bp and 552 bp. Both primer sets gave similar results. qPCR on splenic DNA further confirmed these findings (Figure 4C).

### Detection of EBV in autopsy tissues of rabbits

**Figure 5:**
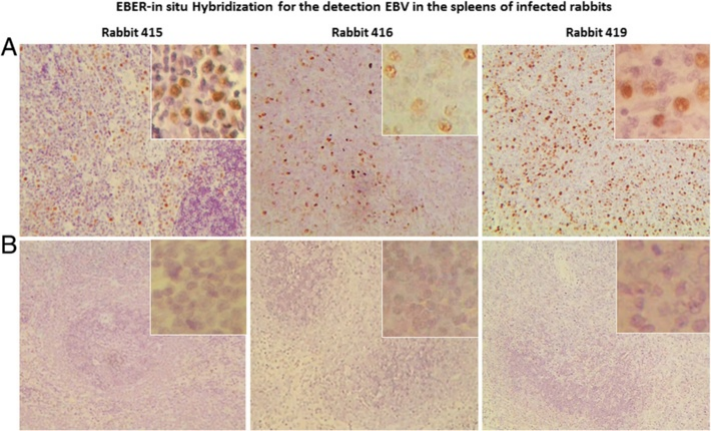
**EBER-** ***in situ*** **hybridization for the detection of EBV in the spleens of infected rabbits.** EBV infected cells were present in the spleens of 3 of the 4 rabbits that survived the 15 day CsA treatment (Panel **A**). Rabbits 416 and 419 had the most extensive infection. EBER-positive cells were heavily distributed in most parts of the spleens of these animals. No staining was seen in the corresponding negative controls (Panel **B**).

EBER-*in situ* hybridization (EBER-ISH) was performed on formalin-fixed, paraffin-embedded sections of rabbit autopsy tissues to detect the virus and its tissue distribution as previously described [43,44]. All major organs were examined, but positive signals were only seen in the spleen and liver of 3 rabbits (Rbt 415, Rbt 416 and Rbt 419) (Figure 5A). No staining was seen in rabbit 420 and in the two rabbits (Rbt 417 and Rbt 418) that died at the start of the CsA treatment. Of the 3 EBER-ISH positive spleens, the most extensive and widespread infection was seen in rabbits 416 and 419 and the least in rabbit 415 (Figure 5A). These observations were consistent with the qPCR findings on PBMCs and spleen tissues from these animals. The negative controls, which consisted of sections from the same spleen, treated and processed at the same time and in exactly the same way, except that non-complementary sense probes were used, consistently gave negative staining (Figure 5B). The EBER-staining pattern was characteristically nuclear with nucleolar sparing, typical of EBER-ISH [43,44].

**Figure 6:**
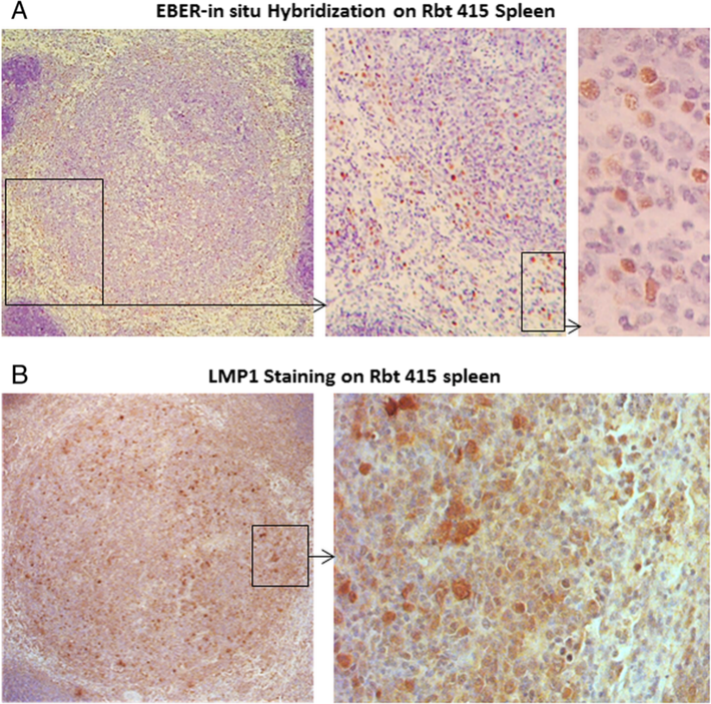
**Detection of EBV and EBV gene expression in a large lymphoid nodule in the spleen of rabbit 415.** In rabbit 415, EBER positive cells in the spleen were localized to one large lymphoid nodule **(A)**. The same nodule was also positive for LMP-1 **(B)**. Both, EBER-*in situ* hybridization and LMP-1 immunohistochemistry were repeated 2 more times on consecutive sections and the same nodule was EBV positive.

Morphologically, the infected cells were large lymphocytes. For rabbit 415, the infected cells in the spleen were restricted to one location which resembled a large lymphoid nodule (Figure 6A). The same nodule was also positive for LMP-1 (Figure 6B). It is possible that this lymphoid nodule is a consequence of EBV-induced proliferation of infected lymphocytes.

**Figure 7:**
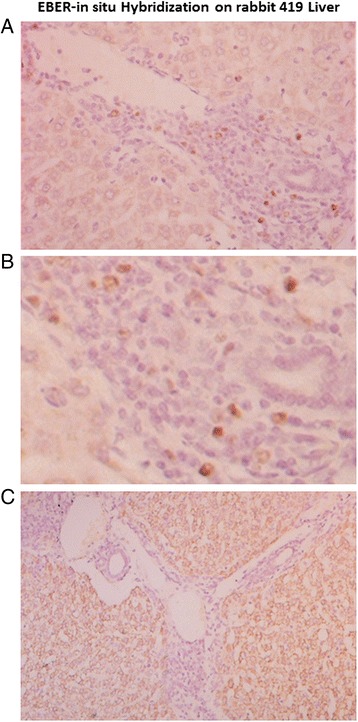
**Detection of EBV in the liver of rabbit 419.** Scattered EBER-positive cells were noted in the liver of all 3 animals that survived the cyclosporin A treatment. However, the EBER-positive cells were limited in number and dispersed within the portal tract regions only and there was no evidence of infection of hepatocytes **(A)**. Morphologically, EBER-positive cells were typically large lymphocytes **(B)**. Negative controls using sense probe were consistently negative **(C)**.

Histologically, the nodule appeared to be of inflammatory nature and not malignant. As for the liver, EBER-ISH positive cells were located almost exclusively in the portal tract regions (Figure 7). There was no histological evidence of any significant inflammation of the liver or infection of hepatocytes in any of the 3 infected rabbits. Rabbit 419 had the most extensive infection. In fact, only scattered EBER-ISH positive cells could be seen in rabbits 415 and 416. Apart from a few scattered EBV-positive cells seen in the lung of rabbit 419, no evidence of EBV infection was noted in any other tissues (lung, heart, kidneys), in any of the other rabbits.

### Detection of EBV gene expression in the spleen and liver of infected rabbits

**Figure 8:**
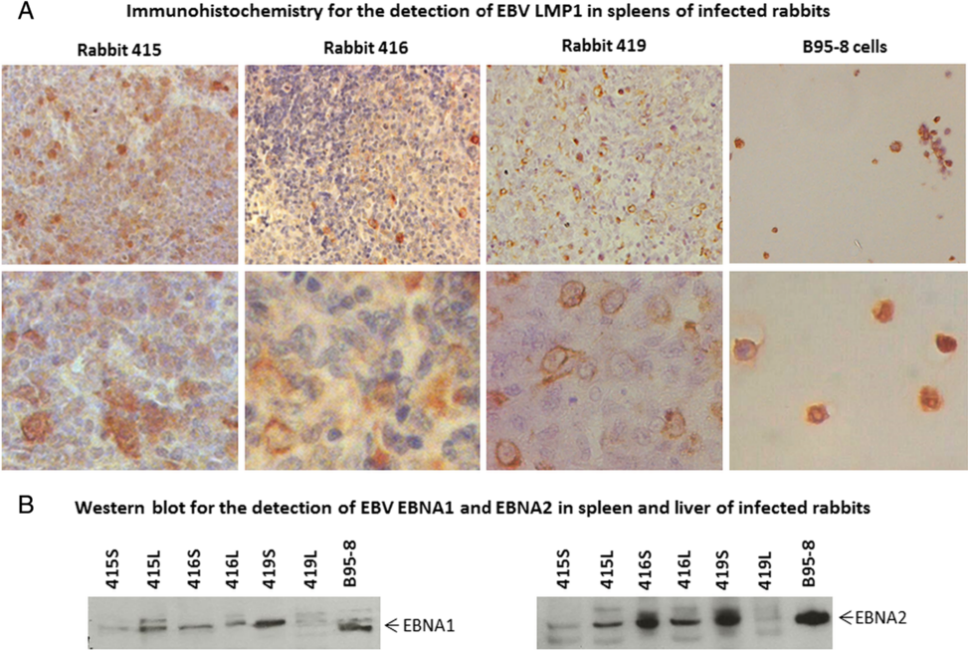
**Detection of EBV expression in the spleen and liver of EBV infected rabbits.** All EBER-positive spleens were tested for the expression of EBV LMP-1 by immunohistochemistry **(A)**. Characteristic membrane and cytoplasmic staining was evident in the spleen of all 3 rabbits **(A)** bottom panel. Formalin-fixed and paraffin-embedded EBV-positive cell line B95-8 was used as positive control. Expression of EBNA-1 and EBNA-2 was examined by western blotting **(B)**. A total of 50 μg of protein lysate was loaded per well. Specific bands were noted in both the liver (L) and spleen (S) of infected animals. Protein extracts from B95-8 cells was used as positive control.

Spleen and liver tissues from the 3 rabbits (Rbt 415, Rbt 416, Rbt 419) which were found to be EBV positive by EBER-ISH were examined for LMP-1 expression using immunohistochemistry. Spleen sections from all 3 rabbits were found to have positive cells (Figure 8A) and they were distributed in the same regions as the EBER-ISH positive cells. Indeed, the one large lymphoid nodule that was EBER-ISH positive in the spleen of rabbit 415 was also LMP-1 positive. The pattern of LMP-1 staining was both membrane and cytoplasmic. Overall, the number of LMP-1 positive cells was distinctly less than the number of EBER-ISH positive cells for the same tissue. In the liver, LMP-1 positive cells were sparsely scattered, even for rabbit 419 (data not shown). Western blotting for EBNA-1 and EBNA-2 revealed that both were expressed in the spleen and liver of EBER-ISH positive animals (Figure 8B). These observations are similar to the RT-PCR results for the expression of these genes in PBMCs (Figure 3B).

## Discussion

It is just over 50 years since EBV was first isolated from a case of Burkitt’s lymphoma. Although much progress has been made over the decades, some basic aspects of EBV biology such as the initial cellular target of primary infection, the cellular source of the virus detected in saliva during persistent infection, and the detailed mechanisms by which this virus induces tumorigenesis is poorly understood. The lack of a suitable laboratory model is certainly one of the contributing factors for the slow progress in these areas. EBV is a highly species specific virus, infecting humans only, with B-lymphocytes being its primary cellular target. Other γ-herpesviruses closely related to EBV have been shown to naturally infect non-human primates [30,45], as well as mice [46,47] and they have been used as models to understand EBV infection and pathogenesis. However, the major drawback with these animal models is that they do not entirely represent EBV infection in humans.

Recently, a Japanese group reported that rabbits (Japanese White) were susceptible to EBV infection and manifest features reminiscent of EBV infection in humans [48,34,36]. In the present study we confirm that healthy rabbits (New Zealand White) can indeed be infected with EBV via intravenous inoculation and the virus persists in peripheral blood lymphocytes of infected animals. We further show that rabbits mount a strong immune response to the virus, as evident by the raised levels of IgM and IgG in the plasma. The immune system is most likely to be the central force responsible for clearing the virus and limiting viral replication and spread. This is supported by our findings of low level viral DNA and little or no gene expression in peripheral blood mononuclear cells (PBMCs) in the 7 week follow up post EBV infection. This is similar to what is generally seen in healthy EBV seropositive individuals [17,18]. However, following immunosuppression with daily injections of cyclosporin A, EBV viral load in PBMCs increased from undetectable levels to tens of thousands of copies by day 15 of treatment. The increase in viral load correlated with increasing duration of immunosuppression. These results were also consistent with qPCR data for EBV viral load in the spleens of the 4 rabbits that survived CsA treatment. Moreover, EBER-ISH revealed the presence of abundant EBV infected cells in the spleens of 3 of 4 rabbits, but not in the two rabbits that died on day 2 of CsA treatment. These findings suggest that under immunosuppressive conditions, EBV infected cells can proliferate, analogous to what has been reported in post-transplant recipients on CsA immunosuppressive therapy [20,24,38,49]. We also noted the expression of LMP-1, EBNA-1 and EBNA-2 in EBV positive spleens. In PBMCs however, the level of expression of some of these genes was weak and intermittent. Previous studies on EBV gene expression in PBMCs from post-transplant recipients have reported conflicting results. Some studies have reported that EBV gene expression is restricted and similar to that seen in healthy carriers [25,50], whilst others have reported that the EBV gene expression pattern is more broader [49,51]. Interestingly, tumor tissues from allograft recipients tend to express type III latency [21-23]. It is possible that EBV gene expression varies at different stages of differentiation of the EBV-infected cells on their route to tumor development [38,52,53]. The pattern of EBV gene expression is also likely to be dictated by the degree and length of immunosuppression [24].

In contrast to the spleen, EBV infection of the liver in these animals was noticeably less. Only scattered EBER-ISH positive cells could be detected and they were restricted to the portal tract regions. There was no evidence of infection of hepatocytes or infiltration of EBV-infected lymphocytes into the liver parenchyma. Morphologically, infected cells resembled lymphoblast. We also noted that there was a correlation between the severity of infection in the spleen and the frequency of infected cells in the liver. Rabbit 419, which had the most extensive infection in the spleen, also had the highest frequency of infected cells in the liver. A few scattered EBV positive cells were also seen in the lungs of rabbit 419. It remains to be demonstrated whether the peripheral circulation or lymphoid tissues is the main site for proliferation of EBV infected cells. Quantitative EBV PCR on PBMCs and spleens at day 15 of immunosuppression showed a higher viral load in the spleens compared to PBMCs in 3/4 rabbits, indicating that lymphoid tissues such as the spleen maybe the preferred site. Furthermore, the finding of abundant expression of LMP-1 in the spleen, but not in the PBMCs of infected animals, also supports lymphoid tissues as the most likely site for EBV proliferation [54]. In the lymphoid tissues, EBV infected cells probably enter the germinal centers where viral and local tissue factors could mediate their proliferation and expansion [55,56]. On differentiation into memory cells expressing little or no viral genes, the infected cells could then enter the peripheral circulation [50]. The rabbit model described here could help to further delineate the dynamics of EBV infection and mechanism of proliferation.

## Conclusion

In conclusion, we confirm that healthy rabbits can be infected with EBV and immunosuppression can induce EBV proliferation similar to what has been observed in transplant recipients. Rabbits may prove to be a convenient and suitable small animal model for the study of EBV infection. For example, determining which cells are first infected following salivary transmission of the virus and the subsequent cellular dynamics leading to the life-long latency typical of EBV would be a significant development in our understanding of the pathophysiology of EBV. Rabbit model could also prove to be useful for evaluating potential therapies and vaccines for the EBV-associated disease [57].

## Methods

### Animals

Six healthy male New Zealand white rabbits (0.8-1.2 kg) aged 2–4 months were purchased from a local animal facility and housed in the Animal House of the College of Medicine and Health Sciences, UAE University in accordance with institutional policies. The project was reviewed and approved by our Institutional Review Board and experiments were performed in accordance with protocols approved by the Animal Research Ethics Committee of UAE University (Approval number A14-12).

### Preparation of EBV for infection of rabbits

B95-8 cells (EBV producer cell line) [2] were cultured in RPMI-1640 (GIBCO, USA), supplemented with 10% FBS (GIBCO, USA), 1% antibiotic antimycotic solution (Santa Cruz, UK), 50 μg/ml gentamycin (Hyclone, USA) and 1× glutamine (GIBCO, USA), as previously described [58]. Cells were cultured until they reached a density of approximately 5×10^6^ cells/ml. Culture supernatants were centrifuged to remove cells/cell debris and then filtered using a 0.45 μm filter. Fresh filtered virus preparations were used for inoculation of rabbits. EBV copy number in the inoculum was estimated using quantitative real-time PCR (qPCR) (see ahead). The infectivity of the virus was determined by *in vitro* immortalization of human PBMCs [40]. This was approved by the Al Ain Medical District Human Research Ethics Committee (Approval number AAMD HREC 14/13).

### Inoculation of rabbits with EBV and immunosuppression

Pre-inoculation blood samples were collected from the ear marginal vein of all rabbits. For EBV infection, animals were injected intravenously with 1 ml of fresh culture supernatant filtrate. EBV inoculation of rabbits was repeated on day 3 and 6 after the first injection. Approximately 4 ml of blood was collected into heparin tubes (BD Bioscience, UK) from each animal on weekly basis for a total of 7 weeks. The heparinized blood was diluted 1:1 in RPMI 1640 media and layered (under sterile conditions) over Histopaque 1083 (Sigma, UK) and centrifuged at 600 g for 20 minutes in a bench top X15R centrifuge (Beckman Coulter, UK). The plasma and peripheral blood mononuclear cell (PBMC) fractions were carefully collected into separate tubes. PBMCs were washed with PBS (x2), cell number determined by trypan blue dye exclusion and then stored at −80°C. Plasma samples were stored at −40°C.

At week 10 post-infection, animals were started on immunosuppressive therapy with daily subcutaneous injection of 20 mg/kg of cyclosproin A (CsA) (Sandimmume -Novaratis) [41,42] for a total of 15 days. Animals were monitored daily by a veterinarian for signs of CsA toxicity, such as drooling, diarrhea, lack of food intake and weight loss [59]. Any animal that became seriously sick was sacrificed in accordance with the ethics guidelines. Blood was collected on days 1, 4, 8, 11 and 15 after commencement of immunosuppressive therapy. On day 17, surviving animals were euthanized and autopsied. Tissue samples from all major organs, including spleen, liver, kidneys, heart and lungs were collected. One piece from each organ was immediately snap-frozen in liquid N_2_ for DNA/RNA extraction and one piece was immediately fixed in 10% formalin for histology, *in situ* hybridization and immunohistochemistry.

### Testing for EBV antibodies in rabbit plasma

EBV specific antibodies in rabbit plasma samples were measured using a modified Enzyme linked immunosorbent assay (ELISA) [60]. Briefly, 96 well ELISA plates (Nunc, Denmark) were coated with proteins from EBV-infected B95-8 cell line. Each well was coated with 100 ng of proteins with overnight incubation. Rabbit plasma at different dilutions (1/100, 1/200, 1/400) was applied to the wells and bound antibodies subsequently detected using goat anti-rabbit IgG (Cell Signaling, USA) or goat anti-rabbit IgM secondary antibody conjugated to HRP (Abcam, UK). After adding the TMB substrate and allowing the colour to develop for 5 minutes in the dark, the reaction was stopped and OD_450_ measured using an ELISA reader (Gen 5 BioTek, USA). All the OD_450_ values were read against the pre-inoculation plasma samples from the same rabbit. All tests were done in duplicates and the mean values were used in further analysis.

### PCR for the detection of EBV in peripheral blood

DNA was extracted from rabbit PBMCs and autopsy tissues using standard phenol-chloroform extraction methodology as previously described [61]. The quantity and purity of the extracted DNA was determined using the Nanodrop-1000 instrument (Nanodrop Technologies, USA). PCR was performed on 100 ng of extracted DNA using primers specific for EBV BamHI W fragment [62]. Amplification of ‘house-keeping gene’ β-globin [63] was performed to assess the integrity of the DNA. Each PCR run of 35 cycles included a positive control (DNA from B95-8 cell line) and a negative control (water). PCR reactions were carried out using an Applied Biosystems thermal cycler GeneAmp® PCR System 2700. Amplified products were visualized on 2.5% agarose gel stained with ethidium bromide.

### Quantitative real-time PCR (qPCR) for determination of EBV viral load

EBV copy number in the initial inoculum and subsequently in the PBMCs/spleen tissues was estimated using qPCR targeting EBV BamH1W region and Namalwa cell line DNA standards, as previously described [39]. Briefly, for each qPCR reaction, 50 ng of template DNA was used in a total reaction volume of 20 μl with ABS TaqMan Universal Master mix along with TaqMan probe. All samples were tested in duplicates or triplicates in a 40 cycle reaction using Applied Biosystem 7500 real time PCR machine. All qPCR experiments were independently repeated 3 times and the mean copy number and standard deviation calculated for each sample.

### EBER-*in situ* hybridization for the detection of EBV in tissues

The presence of EBV in autopsy tissues was determined by using a very sensitive and highly specific technique of EBER-*in situ* hybridization, essentially as previously described [43,44]. Briefly, EBER-*in situ* hybridization was performed on 5-μm sections of formalin-fixed, paraffin-embedded tissue sections using a mixture of digoxigenin-labeled EBER-1 and EBER-2 probes with overnight hybridization. Hybridized probes were subsequently detected using mouse anti-digoxin monoclonal antibody at a dilution of 1/2500 (Sigma, UK) and the ABC-peroxidase method (Ultra-Sensitive ABC-Peroxidase Staining kit, Thermo Scientific, USA). Diaminobenzidine tetrahydrochloride (DAB) (Sigma, UK) was used as the chromogen. With each batch of tissue sections, a positive control (EBV-infected B95-8 cells or EBV-lymphoblastoid cell lines) and a negative control (using digoxigenin-labeled non-complimentary EBER probes) was included.

### Reverse transcription (RT)-PCR for the detection EBV gene expression in PBMCs

Total RNA was extracted from rabbit PBMCs using TRIzol reagent (Invitrogen) and quantified using the Nanodrop instrument. 1 μg of RNA was reverse transcribed to cDNA using the Reverse Transcription System (Promega) following the manufacturer’s instructions. Before reverse transcription, all RNA samples were treated with DNase I (Promega, USA) to remove any contaminating genomic DNA. RT-PCR was performed to determine the expression of EBNA-1, EBNA-2, LMP-1 and BZLF-1 using primers and conditions previously described [64]. Amplification of ‘housekeeping gene’ GAPDH was used as an internal positive control [65]. All RT-PCR reactions consisted of 35 cycles with a final elongation at 72°C for 10 minutes.

### Immunohistochemistry and western blotting for the detection of EBV gene expression

Expression of EBV LMP-1, EBNA-1, and EBNA-2 in rabbit tissues was assessed by immunohistochemistry and/or western blotting. Immunohistochemistry was performed on 5 μm sections of formalin-fixed paraffin embedded rabbit spleen and liver tissues using LMP-1-specific monoclonal antibodies (clone CS1-4) (Abcam, UK) at 1/100 dilution and the ABC-peroxidase/DAB detection system. Western blotting was performed on 50 μg of protein extracts from rabbit spleen and liver tissues. The following monoclonal antibodies were used: anti-EBNA-1 (clone 0211 - Thermo Scientific) and anti-EBNA-2 (clone PE2 - Abcam) at dilutions of 1/50 and 1/200 respectively.
